# High-speed video recordings of metal powder pneumatic conveying in thin capillary pipes

**DOI:** 10.1038/s41597-025-04515-w

**Published:** 2025-02-05

**Authors:** Lorenzo Pedrolli, Luigi Fraccarollo, Beatriz Achiaga, Alejandro Lopez

**Affiliations:** 1 University of Deusto, Faculty of Engineering, Bilbao, 48007 Spain; 2 University of Trento, Department of Civil, Environmental and Mechanical Engineering, Trento, 38123 Italy

**Keywords:** Imaging techniques, Characterization and analytical techniques

## Abstract

Many industrial processes require a consistent material flow in the form of powders, typically achieved through pneumatic conveying. This work presents a dataset of high-speed video recordings capturing the horizontal pneumatic conveying of metallic powders in thin capillary pipes. Additionally, pressure and photodiode recordings are included. The experimental setup is described in detail, with suggestions for potential improvements. In the video recordings, each particle is individually distinguishable and can be tracked across frames.

## Background & Summary

Pneumatic conveying systems play a vital role in various industrial processes, enabling the efficient transport of powdered materials. Unlike gravity-fed systems, these systems utilize airflow to move particles through pipelines. This study focuses on the horizontal pneumatic conveying of metallic powders in thin pipes and provides a detailed dataset of high-speed video recordings.

The experimental setup features a high-speed camera positioned to capture the flow of metallic powders through transparent capillary pipes. Thin capillary pipes are ideal for visualizing individual particles, which can be tracked frame by frame. These high-speed video recordings offer detailed insights into flow dynamics, particle interactions, and potential clustering within the conveying system.

The metallic powders used in the study are characterized by their particle size distribution, shape, and density, as these factors significantly influence the flow behavior. The airflow rate is carefully controlled to maintain a consistent conveying environment. Various flow rates and powder feed rates are tested to capture a range of conveying conditions, providing a comprehensive dataset for analysis.

Previous studies on pneumatic conveying have primarily focused on time-averaged flow rates and their applications in specific industrial processes, such as additive manufacturing techniques like Laser Metal Deposition (LMD). Research by Mezhericher *et al*.^1^ and Baraldo *et al*.^2^ has documented phenomena such as pulsating flows and periodic regimes in larger-scale systems, while studies by Zhao^3^ have explored particle motion and velocity in horizontal channels using computational simulations. Despite these contributions, there is a critical need for high-resolution, real-time visualization of particle flow in confined geometries, such as capillary pipes.

Datasets like this are essential for validating computational models. We compared Eulerian-Lagrangian models such as CFD-DEM and MP-PIC^4^, finding significant differences in their representation of particle clustering and flow irregularities. However, the accuracy of such models often cannot be assessed without the appropriate experimental data, underscoring the value of high-quality empirical datasets like the one presented here.

This dataset promotes multidisciplinary research and supports a better understanding of particle dynamics in confined pneumatic systems. By enabling the study of phenomena such as clustering, segregation, and flow irregularities, it provides valuable insights for improving system designs and addressing operational challenges across a range of industries. These insights are particularly relevant for advanced manufacturing processes, such as powder bed fusion and binder jetting, where understanding powder behavior is critical for achieving uniform material deposition and optimizing product quality.

Beyond manufacturing, the dataset has potential applications in microfluidic systems, where confined particle flows in narrow channels exhibit dynamics similar to those observed in capillary pipes. It is also valuable for pharmaceutical processes involving fine powders, such as precise dosing and mixing. These scenarios highlight the broader utility of this dataset for improving efficiency and performance in diverse industrial contexts.

This dataset complements prior studies on pneumatic conveying, such as Santo *et al*.^5^, which employed laser Doppler velocimetry (LDV) to investigate particle concentration and velocity distributions in horizontal conveying of pulverized coal. Unlike coal, the metallic powders studied here exhibit distinct properties, such as higher density and unique particle size distributions, which influence their behavior in conveying systems. While LDV provides precise point-based measurements, our high-speed video recordings offer a spatial and temporal perspective, enabling visualization of particle trajectories and interactions in confined geometries.

This dataset addresses a critical gap in the literature by offering granular-level data that enables the study of clustering, segregation, and flow irregularities in confined geometries and dilute flow. Unlike time-averaged measurements, the frame-by-frame tracking capability allows for a detailed analysis of particle dynamics, particularly under varying flow conditions. These insights are directly applicable to advanced manufacturing processes, such as powder bed fusion and binder jetting, where understanding powder flow behavior is crucial for achieving uniform material deposition and optimizing product quality.

## Methods

Experiments were conducted with the three pipes at different gas flowrates, and using different materials, resumed in Table 1. The total number of combinations is 60.

**Table 1:** Variables changed during the experiments. Combining all of them results in 60 different conditions.

Pipes	Materials	Inlet flowrate [L/min]
Circular *φ*1.15 mm ◯	MetcoAdd 316L-A	0.2
Square 1 × 1 mm □	MetcoAdd 316L-D	0.4
Rectangular 6 × 0.6 mm	MetcoClad 718	0.6
	Titanium Ti6Al4V	0.8
		1.0

For the rectangular pipe, the lowest flowrate (*V* = 0.2L/min) was omitted, since the low gas velocity (*μ* = *V*/*S*_*rect*_ = 0.831 m/s, where *S* is the hydraulic section) was not sufficient to initiate the dilute phase transport of the powder. In this case, the pipe would partially clog due to the insufficient gas velocity, until enough of the hydraulic section was occupied by a stationary bed. This work, however, is focused on dilute flow conditions.

### Conveying circuit

**Fig. 1:**
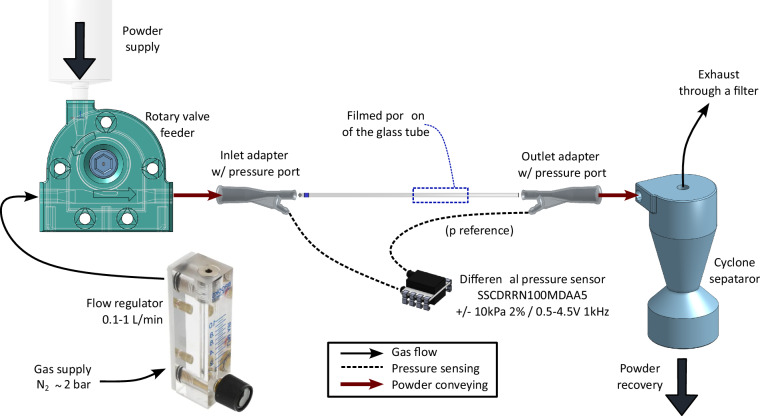
Schematic of the pneumatic conveying circuit used in the experiment.

The pneumatic conveying circuit is presented in Fig. 1. The key part of the system is the borosilicate glass pipe, whose wall thickness is $\mathrm{0.2\ }{\rm{mm}}$ in all cases. This setup can employ three different shapes:

**Circular**, with an internal diameter of 1.15 mm, 75 mm long;

**Square**, with a passage section of $1\times \mathrm{1\ }{\rm{mm}}$, relatively sharp corners, and 100 mm long;

**Rectangular**, with a passage section of $6\times \mathrm{0.6\ }{\rm{mm}}$, with a complete rounding of the short sides, and 100 mm long

**Fig. 2:**
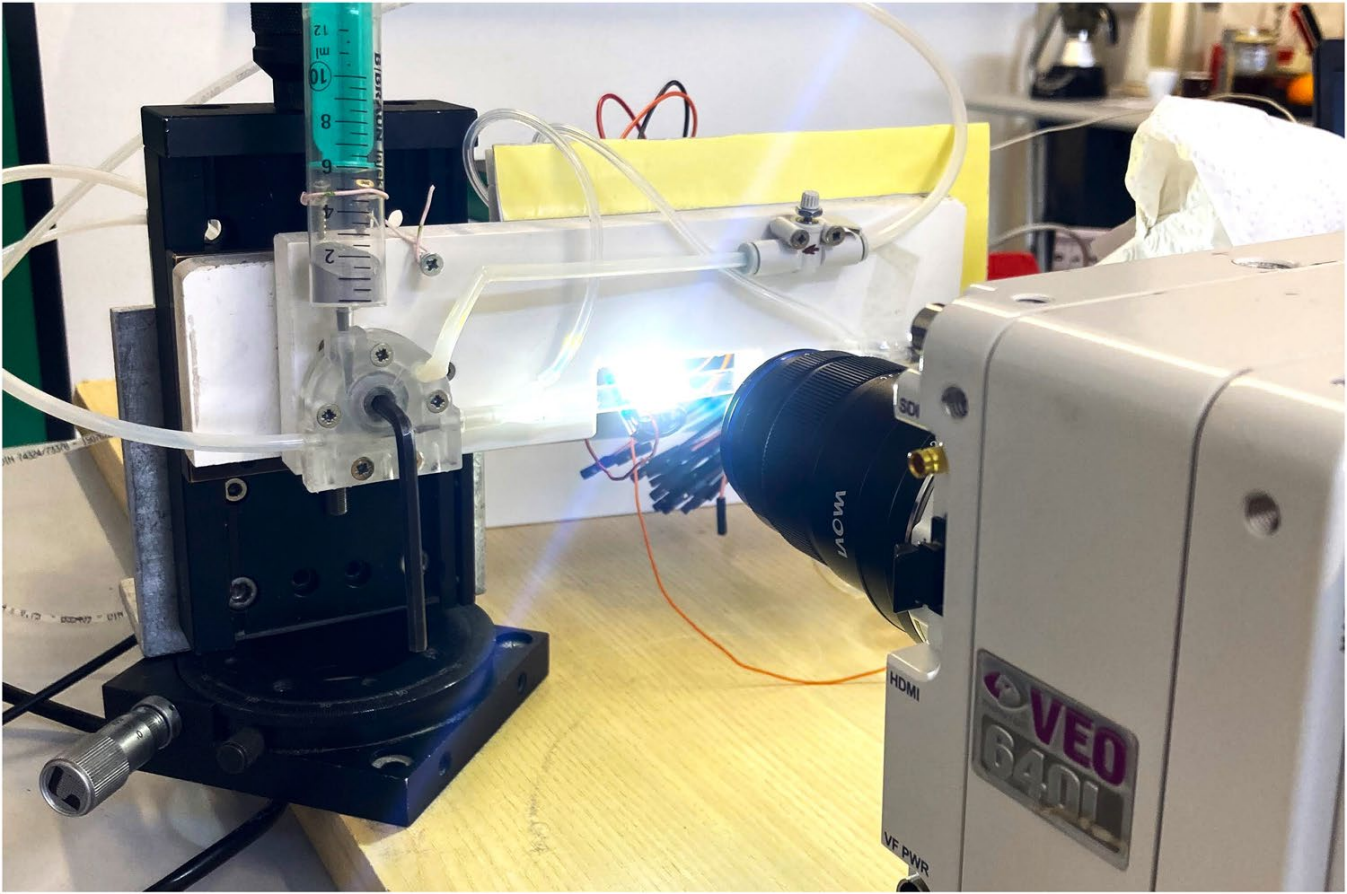
Photo of the setup for the pneumatic conveying experiment.

The setup is captured in the picture of Fig. 2, where also the camera and lighting system are visible. Notice the vertical adjustment of the whole pipe, other adjustments were done by strategic placement of shims and adhesive tape.

#### Glass pipe couplers

**Fig. 3:**
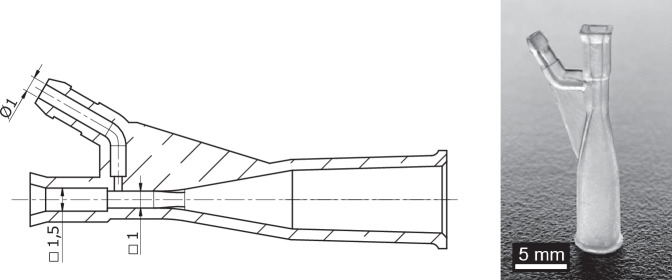
One of the couplers, used for the square pipe. The opening of the static pressure port in the manufactured part is barely visible to the naked eye. A section view presents the internal channels. The conic section is used to fit the coupler to the flexible pipe.

The development of the system involved the design of the small-scale couplers for the pipe. This task was performed by using a Stereolithography (SLA) 3D printer, which allows to produce strong and dense parts with very fine details. The 3D model dimensions were adjusted to obtain the desired fit of the parts. Careful part orientation allowed to manufacture the static pressure measurement ports, which are only a fraction of a mm wide, and any resin trapped in it would cause the opening to clog shut and the part to fail. Figure 3 shows the finished result.

The same technology allowed to manufacture and iterate on a rotary valve feeder. This element is the weak point of the setup and needed to be substituted every few minutes due to excessive erosion, and it is one interesting point of improvement, but it allowed to effectively feed the powder in the circuit for the short duration of the measurements. The main body presents a powder inlet and a wheel, which transports the powder towards the bottom of the component. There, a small venturi accelerates the gas flow, picking up the powder and sending it downstream through the glass pipe.

The gas supply is provided by a high-pressure tank of clean, dry, pure nitrogen gas. Two line regulators step down the pressure first from ∼200 bar to 8 bar, and a second regulator further steps it down to the pressure used in the setup (≤2 bar). This high purity gas supply ensures that the contamination from moisture or oils is not present, and completely avoids a compressor which might send vibrations down the gas line. A needle regulator was used to set the gas flowrate between 0.1 and 1L/min.

#### Sensors integration

The circuit schematic in Fig. 1 shows that the pressure ports are connected to a Honeywell SSCDRRN100MDAA5 (https://sps.honeywell.com/us/en/products/advanced-sensing-technologies/healthcare-sensing/board-mount-pressure-sensors/trustability-ssc-serieshttps://sps.honeywell.com) piezo-resistive differential pressure sensor. The sensor provides a 0.5–4.5V analog output, with a full scale of ±10 kPa. The 3D-printed couplers, see Fig. 3, were designed to have a lateral port to measure the static pressure at the inlet and outlet of the pipe. A batch of couplers was printed, selecting those that provided best alignment of the central channel between the glass pipe and the coupler. Therefore, minimum disturbance is expected when measuring the static pressure.

The calibrated output values are updated at 1 kHz, and recorded using a 14-bit USB oscilloscope. The sensor provides an accuracy of ±0.25% FSS (Full Scale Span) in the same measurement period. When accounting for other effects (temperature variation, voltage offset, calibration, orientation respective to gravity), the manufacturer declares a total error band of ±0.2% FSS. Consequently, while the absolute value of the differential pressure can be determined within ±0.2 kPa, the accuracy for tracking pressure changes over time remains at ±0.025 kPa.

The second channel of the oscilloscope was connected to a photodiode, which has vastly superior response times (μs or lower) compared with the more common photoresistors (several ms). The photodiode is a Vishay BPW34, with a sensing area of 3 × 3 mm, and ability to sense visible light (but the sensitivity peak is in the infrared, at 900nm). A simple voltage divider and amplifier was used as preconditioning circuit. The same background lighting used for the video recording shines through the pipe and illuminated the photodiode. The recorded voltage value is lower with a decrease in luminosity, which is proportional to the amount of particles present in the section of pipe in front of the sensor at any given instant. The absolute voltage value varies between the recordings due to the measurement conditions, it is advisable to use relative values.

### Camera configuration

A https://www.phantomhighspeed.com/products/cameras/veo/veo640Pantom VEO 640 L high speed camera is shown in the setup of Fig. 2. The camera is equipped with a CMOS sensor featuring a global shutter, ensuring uniform and synchronized exposure across all pixels. It offers a resolution of 2560 × 1600 pixels, with each pixel being a square of side 10 μm.

The camera’s electronic global shutter allows for independent control of exposure time and frame rate. Under good illumination conditions, an exposure time of approximately 10 μs was found to provide the best balance between brightness and motion clarity. Longer exposure times improve brightness but cause fast-moving particles to appear as streaks.

The lens is a *Laowa 25 mm f/2.8 2.5-5X Ultra Macro* lens. With its f/2.8 aperture, the lens allows significant light transmission to the sensor, a critical feature for high-speed video capture where fast shutter speeds reduce exposure time. The aperture can also be stopped down to f/16 to increase the depth of field, albeit at the expense of reduced light reaching the sensor. Given the camera’s sensor size of 25.6 × 16 mm (circa APS-C), pixel size of 10um. The recording window for the circular and square pipes was selected at 1280 × 400 px, with a sample rate of 10 000 fps. At full acquisition window, the Field Of View (FOV) is reported in Table 2.

**Table 2:** Resulting field of view and pixel size for the camera setup at minimum and maximum magnifications.

Magnification	Field of View	Pixel Size
2.5x	10.24 × 6.4 mm	4um
5x	5.12 × 3.2 mm	2um

**Fig. 4:**
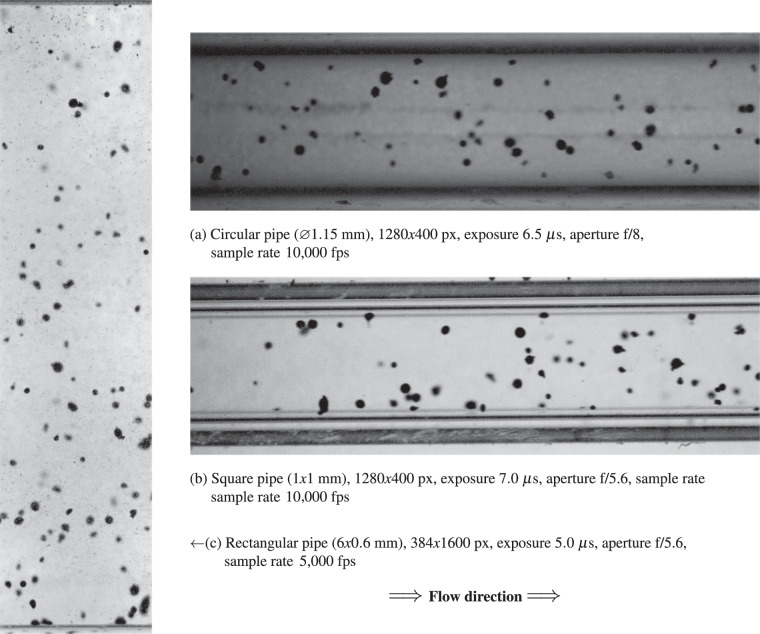
Images captured with Phantom VEO 640 L high-speed camera and Laowa ultra-macro lens for circular (**a**), square (**b**), and rectangular (**c**) pipe sections.

The camera is also mounted on a slider, which allows it to move closer or farther from the pipe in a precise manner, changing the focus. Especially with full aperture, the field of view is quite shallow, and it is crucial to put the focus plane on the center of the pipe. The pipe assembly is moved in order to align its mid-plane with the recording plane. The image is captured around the middle of the pipe length, limiting edge issues. The resulting pictures are of good quality and an example with minimal post-processing is shown in Fig. 4.

**Fig. 5:**
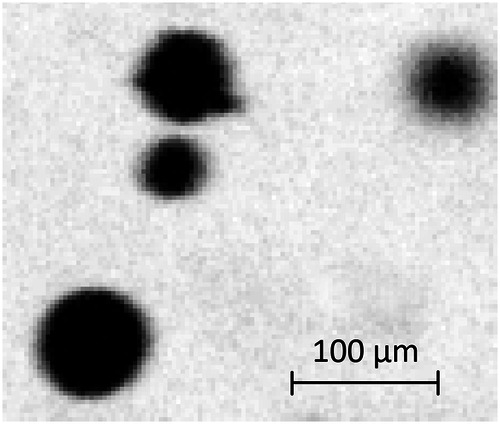
Detail view of four particles captured in Fig. 4(b).

Figure 5 presents a detailed view of a group of medium-sized particles, where the pixel size is approximately $\mathrm{3\ }{\rm{\mu }}{\rm{m}}/{\rm{px}}$. In the absence of a dedicated calibration target, we calibrated the pixel size using the internal dimensions of the capillary pipes, as reported in Table 1. The internal walls of the pipes are visible in the recordings, and the provider specifies a tolerance interval of $\mathrm{10 \% }$ on their internal diameter.

The image resolution enables a direct estimation of the particle size distribution (PSD). The observed PSD closely matches the expected particle diameter, approximately $\mathrm{100\ }{\rm{\mu }}{\rm{m}}$, which supports the adequacy of the calibration method. According to the manufacturer’s indications, the particles are expected to be mostly spherical. Particularly large particles may represent clusters rather than individual particles. While such cases were not analyzed in detail, irregular or misshapen shadows could qualitatively suggest the presence of clusters.

Illumination of the section of interest is directly from the side opposite from the lens. A high powered LED light shines light towards the pipe and to the lens. It is extremely bright, and it is advisable to use some level of PPE when on. A layer of frosted acrylic sheet in front of the LED diffuses the light, resulting in sharper images and a more uniform background.

The LED array is powered in constant current mode, at 800 mA and around 12 V, for a total power of 10 W. The simple circuit uses a common LM317 linear regulator, and a $\mathrm{1.5\ }\varOmega$ resistor. In this way the LED does not present any flicker, which would be picked up by the high speed camera.

### Motion Blur and other challenges

Motion blur is a common challenge in video recording, particularly in experiments involving fast-moving objects. It occurs when the object travels a significant distance during the exposure time, resulting in a visible streak in the recorded image. The extent of the streak is directly proportional to both the velocity of the object and the exposure time of the camera sensor.

In our experimental setup, we carefully optimized the exposure time to minimize motion blur while ensuring adequate image brightness. The exposure time was set to approximately $\mathrm{10\ }{\rm{\mu }}{\rm{s}}$, which was sufficient to minimize streak formation while maintaining an acceptable signal-to-noise ratio and proper image illumination with an aperture setting between f/5.6 and f/8.

The relationship between particle velocity, exposure time, and the resulting motion blur can be estimated mathematically. For example:

- At a velocity of 10 m/s and an exposure time of 10 *μs*, a particle moves approximately 100 *μm* during exposure, causing a visible streak.
- At a velocity of 3 m/s and an exposure time of 1 *μs*, the particle displacement is reduced to only 3 *μm*, rendering the streak nearly imperceptible.
- Under our typical experimental conditions, with an average velocity of 4 m/s and an exposure time of 7 *μs*, the expected displacement is approximately 28 *μs*. However, due to the Gaussian nature of the blur, the streak’s tails are less pronounced, making the blur less visually intrusive.
  It is important to note that motion blur was primarily assessed visually in this study, as a detailed quantitative analysis of blur was outside the scope of our investigation. Nonetheless, minimizing motion blur requires balancing several interconnected parameters, including:
- Exposure time: Shorter exposure reduces streak length but may reduce image brightness.
- Illumination intensity: Sufficient illumination compensates for shorter exposure times.
- Aperture size: Optimized to balance light collection and depth of field.
- Focus depth: Ensuring particles remain sharply focused within the field of view.
- Reflections and diffraction effects: These optical phenomena can contribute to image artifacts if not managed carefully.
  In conclusion, the achieved exposure time of approximately 10 μs provided a reasonable compromise between minimizing motion blur and maintaining sufficient image brightness. This setup enabled us to capture clear and detailed images of individual particles in motion, supporting accurate particle size distribution (PSD) analysis and flow characterization.
  To adapt the same technique used to obtain this dataset to additional application scenarios, potential modifications may include:
- Using higher magnification lenses for smaller particles, this requires to increase the light concentration.
- Using telecentric lenses for better size accuracy, especially in a deeper field of view.
- Employ filters or polarized light to improve the visibility of translucent or transparent particles.
- Altering pipe materials and geometries to more closely replicate conditions found in specific processes.

## Data Records

The files are available on Zenodo^6^ (10.5281/zenodo.12938843).

The published dataset contains a total of 101 video recordings in *.mp4 format, and each comes with a *.csv file of the same name. The recordings are organized in four different archives, divided by material.

All images have a name in a predefined format sequentially, with the pattern:

<Material> _ <pipe size> _ <gas flowrate> _ <test number> _ <optional> . <extension>. For the first three keys refer to Table 1, which reports the material name, the pipe sizes and flowrates.

Some videos are repeated in similar conditions, and this is denoted by test01, test02 and so on.

The optional key can indicate that the video recording was saved at a different framerate. For instance, decimated10x means that the framerate is 10 times lower, or if the original video is 10 000 fps, the decimated one is 1 000 fps.

In addition to video data, there are pressure drop and luminosity recordings (by the photodiode) that can be synchronized with the corresponding video feed, providing a comprehensive dataset. Both pressure and luminosity values are recorded in a CSV file. The luminosity is recorded in volts (V), and this value is correlated to the amount of particles present in the pipe between the background light and the photodiode. The optional key in the CSV files determines the pressure sensor used, indicating its range: either 1 kPa or 10 kPa, which is also recorded as raw data in volts (V).

In order, the columns in all the CSV files are as follows:

- Time [s], Pressure [V], Luminosity [V]

## Technical Validation

The high-speed video recordings were meticulously analyzed to ensure clarity and accuracy in capturing particle flow. The clarity of the images, facilitated by the high-quality camera and lighting setup, was validated by visually confirming that individual particles could be distinctly identified and tracked across frames.

Pressure drop and luminosity values were recorded in a CSV file, synchronized with the video feed. The sensor’s manufacturer declares an accuracy of $\pm \mathrm{0.25 \% }$ FSS and total error band of $\pm \mathrm{2 \% }$ FSS. The luminosity values, recorded in volts (V) using a Vishay BPW34 photodiode, were correlated with the amount of particles in the pipe, visible through the high-speed camera.

**Fig. 6:**
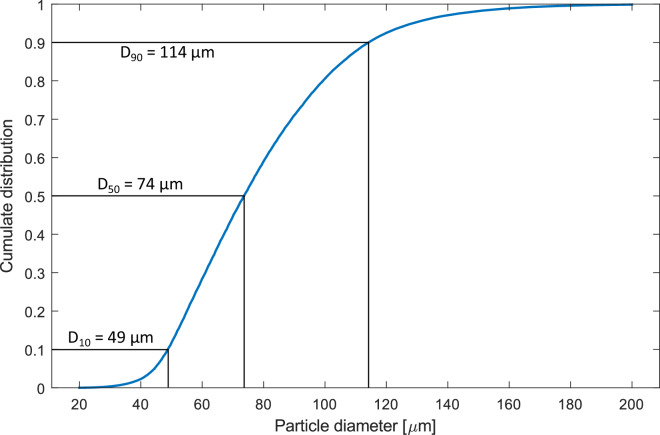
Particle Size Distribution (PSD) measured by the Particle Tracking (PT) algorithm.

The video recording was calibrated using the visible pipe diameter as calibration ruler. The suggested open-source code Fiji/ImageJ^7^ was used to determine the size of the particles visible across the recording. The accuracy of this measurement is very dependent on the sharpness of the original images, and on the parallax error. The camera setup can be improved by a telecentric lens, and secondarily by a parallel rays light source. Nonetheless, the PSD shown in Fig. 6 was determined in the recording conducted using a circular pipe of *d* =  1.15 mm at flowrate of *V* =  L/min. The material is AISI 316 L stainless steel, in the commercial form *Oerlikon MetcoAdd 316L-D* with a nominal PSD *D*_90_ = 106 μm. The measured PSD is very close to the values declared by the manufacturer of the powder.

## Usage Notes

The videos captured using the described setup can be analyzed as an “image stack,” or a sequence of 8-bit gray scale images, in the image processing software Fiji/ImageJ^7^. In the pre-processing step, the original video can be rotated, cropped, and transformed into a black and white binary mask according to a brightness threshold. After loading the video and making sure it is 8 bit black and white, image processing can be performed with the example macro provided in the repository^6^.

More advanced image filtering and segmentation methods are available in the software and can be tested, but basic pre-processing could be enough thanks to the camera and lighting setup, which provide easily identifiable spots. In this context, particles refer to the physical 3D objects that compose the powder, while spots are their 2D shadows captured in each image of the high-speed video.

Particle tracking and analysis can be performed using TrackMate^8^, an additional plugin to Fiji. Essential to this work is its implementation of the linear motion tracker, which can handle particles moving with a roughly constant velocity. It is based on the LAP framework^9^, which approximates multiple-hypothesis tracking using a linear assignment problem: a cost matrix penalizes the least likely movements. Originally developed to track the Brownian motion of bacteria, TrackMate uses this method to track fast-moving objects with a Kalman filter strategy. Conveniently, the spot identification step of TrackMate records the geometric descriptors of each spot: area and perimeter are used to calculate the equivalent diameter, along with other shape descriptors like circularity, Feret diameters, and ellipsoid axes.

### Pressure sensor

The pressure values are recorded in volts (V) and can be converted to pressure units using the conversion factors provided by the manufacturer in the sensor’s datasheet (https://sps.honeywell.com/us/en/products/advanced-sensing-technologies/healthcare-sensing/board-mount-pressure-sensors/trustability-ssc-serieshttps://sps.honeywell.com). The differential pressure sensor has a range of either $\pm \mathrm{1\ }{\rm{kPa}}$ or $\pm \mathrm{1\ }{\rm{kPa}}$. The file name indicates which sensor was used: 1 kPa or 10 kPa.

In the case of the $\pm \mathrm{1\ }{\rm{kPa}}$, the pressure drop ($\varDelta p=0\,{\rm{Pa}}$) corresponds to $\mathrm{2.5\ }{\rm{V}}$. The nominal full range is from $\mathrm{10 \% }$ to 90% of ${V}_{{\rm{supply}}}=5{\rm{V}}$, therefore from $500{\rm{mV}}$ to $\mathrm{4500\ }{\rm{mV}}$. The conversion is linear with a coefficient ${K}_{1}=\mathrm{2000\ }{\rm{mV}}/{\rm{kPa}}$. For example, a value of 2.789 V corresponds to a positive differential pressure of 0.1445 kPa. A value of 1.354 V corresponds to a negative differential pressure of −0.5730 kPa.

For the $\mathrm{10\ }{\rm{kPa}}$ sensor, the conversion parameter is ${K}_{10}=\mathrm{200\ }{\rm{mV}}/{\rm{kPa}}$.

### Luminosity sensor

The photodiode was amplified to provide a signal in the 0-5 V range. The ambient luminosity and sensor positioning suffered inevitable shifts during the day, but is consistent within a single measurement. The values are expected to directly correlate with particle concentration; however, comparison with the high-speed camera video data is required.

## Data Availability

This work did not use custom code. One option to perform the post-processing is the TrackMate plugin^8^. A modified version of the plugin useful to analyze the presented dataset is available at this URL: https://github.com/trackmate-sc/TrackMate/pull/296.
